# Correlation between perinatal asphyxia-induced brain injury, number of enlarged perivascular spaces, and sleep quality in later life

**DOI:** 10.1007/s12519-026-01093-8

**Published:** 2026-09-29

**Authors:** Xin-Yu Xue, Li Sun, Min Yu, Mei-Xia Li, Qiu-Yan Tian, Jing Ren, Po Miao, Li-Xiao Xu, Chen-Xi Feng, Mei Li, Min Gong, Ling-Zhi Wang, Hong-Liang Huo, Jian Huang, Wan-Liang Guo, Rong Ju, Tao Pan, Xing Feng, Wen-Hao Zhou, Huai-Yu Li, Bin Sun, Xiao-Feng Yang, Xin Ding

**Affiliations:** 1 Soochow Key Laboratory of Prevention and Treatment of Child Brain Injury, Children’s Hospital of Soochow University, Suzhou, China; 2 Department of Pediatrics, Richmond University Medical Center, New York, USA; 3 Department of Neonatology, Taizhou Municipal Hospital, Taizhou, China; 4 Department of Rehabilitation, Children’s Hospital of Soochow University, Suzhou, China; 5 School of Basic Medical Sciences, Medical College of Soochow University, Suzhou, China; 6 Department of Radiology, Children’s Hospital of Soochow University, Suzhou, China; 7 School of Medicine, Chengdu Women’s and Children’s Central Hospital, Chengdu, China; 8 Guangzhou Women and Children’s Medical Center, Guangzhou Medical University, Guangzhou, China; 9 Department of Neonatology, General Hospital of Ningxia Medical University, Yinchuan, China

**Keywords:** Children, Enlarged perivascular spaces, Pediatric, Perinatal asphyxia, Perinatal asphyxia-induced brain injury, Sleep quality

## Abstract

**Background:**

Perinatal asphyxia (PA) is increasingly recognized as a significant contributor to neonatal brain injury. Identifying reliable biomarkers for assessing the severity of injury is crucial. Enlarged perivascular spaces (EPVSs), which are considered imaging markers of glymphatic dysfunction are reliable indicators of disease severity and prognosis. However, the application of EPVSs among neonates remains unclear. This study aims to investigate whether numbers of EPVSs can serve as a novel biomarker for the severity of PA-induced brain injury (PABI) and the sleep quality in later life.

**Methods:**

Neonates (*n* = 319) with PA were categorized as mild (*n* = 229) or severe (*n* = 90). Clinical, amplitude-integrated electroencephalography (aEEG), and cranial magnetic resonance imaging (MRI) data were collected. The sleep quality was assessed using questionnaires via follow-up. Correlation analysis was performed to examine associations between numbers of EPVSs and clinical parameters. Multivariate linear regression analysis was performed to evaluate the independent correlation between numbers of EPVSs and severity of PABI. The correlation between numbers of EPVSs and later sleep quality in infants was also evaluated.

**Results:**

There were multiple clinical parameters correlated with numbers of EPVSs. Univariate analysis revealed that physiological status, inflammatory markers, and numbers of EPVSs were related with the severity of PABI. Multivariable regression analysis found that numbers of EPVSs independently related with the severity of PABI [*P* = 0.0001 in all infants (MRI); *P* = 0.0248 in mild PA group (MRI); *P* = 0.0114 in severe PA group (MRI)]. In the severe PA group, numbers of EPVSs were significantly correlated with long-term sleep quality (*P* = 0.0061, *r* = 0.3365), a higher number of EPVSs was associated with more severe sleep disturbance.

**Conclusions:**

Numbers of EPVSs may serve as a novel imaging biomarker for evaluating the severity of brain injury after PA; this biomarker can predict sleep disorders later in life. Collectively our data suggests that glymphatic dysfunction may contribute to post asphyxia-induced brain injury. Early evaluation of EPVSs could aid in risk stratification and intervention strategies.

**Graphical abstract:**

**Figure:**
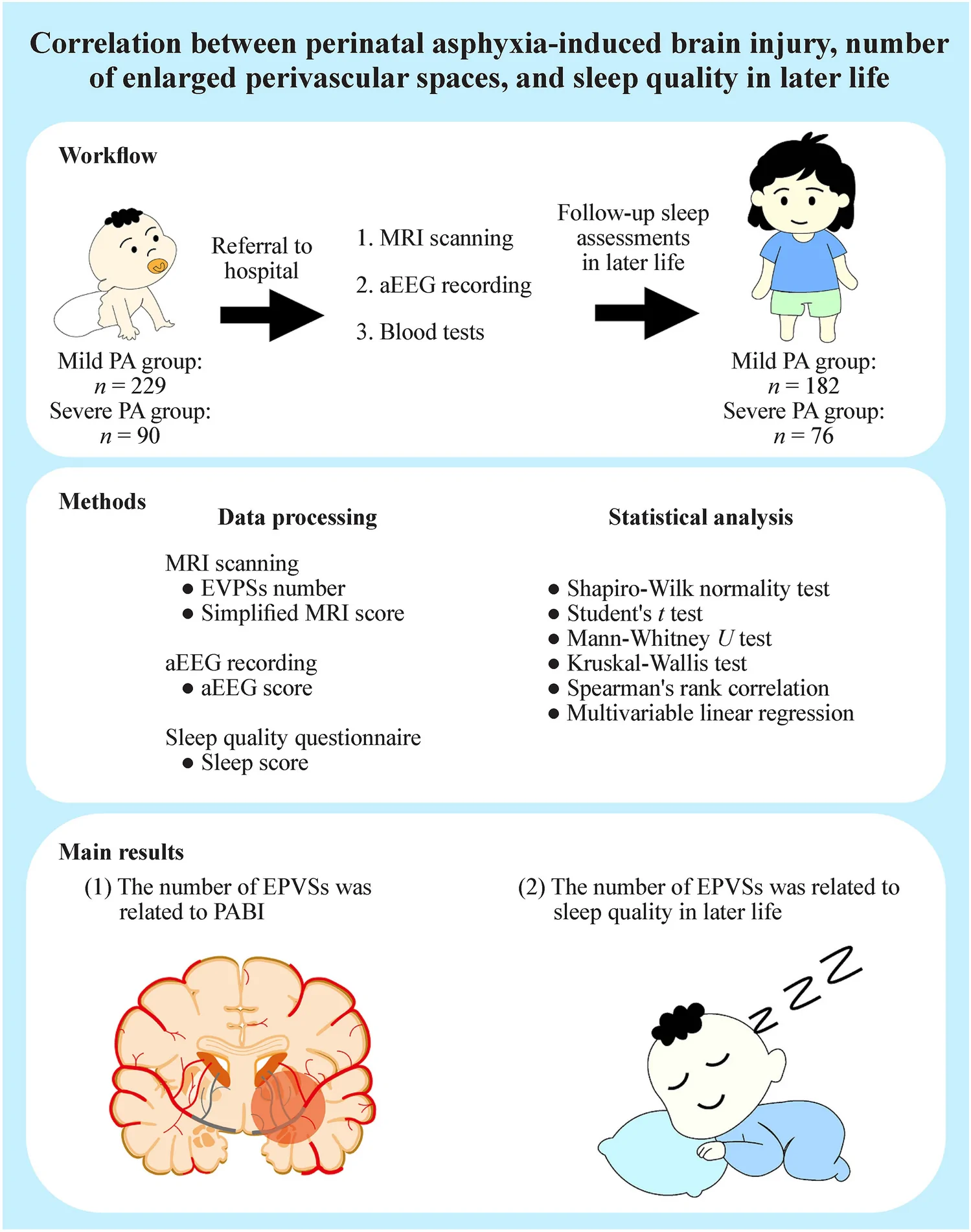

## Introduction

Perinatal asphyxia (PA) is a common and complex neonatal disease with a high incidence and a wide range of sequelae [1]. The neonatal brain is underdeveloped and extremely sensitive to hypoxia [2]. PA can cause significant neurological damage through mechanisms that include oxidative stress and cytokine storm [2, 3]. Regardless of disease severity, PA is associated with a range of long-term neurological sequelae. Mild PA-induced brain injury (PABI) may damage the pineal gland, potentially contributing to sleep disorders and neurodevelopmental impairment [4–6]. Severe PABI can progress to hypoxic ischemic encephalopathy, causing brain dysfunction and severe outcomes such as cerebral palsy [7–9]. These sequelae not only seriously affect the child’s quality of life but also impose a heavy burden on families and society. Advancements in neonatal diagnostic technologies have increased the recognition of PA-induced brain injury but there is an urgent need for novel diagnostic and prognostic targets [10].

Most current biomarkers for PABI focus on inflammatory factors or neuron-specific enzymes. These biomarkers have limitations, including a lack of specificity and an inability to directly reflect intracerebral pathological processes [11–13]. In recent years, enlarged perivascular spaces (EPVSs) have emerged as a novel imaging biomarker and attracted increasing attention because of their significance with various neurological disorders [14, 15]. Normal structure of the PVS is the foundation for normal glymphatic function [16, 17]. Several studies have shown that a greater number of EPVSs and larger EPVS volume are associated with worse glymphatic function and thus reflect overall brain health. Moreover, these indices facilitate evaluation of disease severity in adult patients and may also predict patient prognosis [17]. However, EPVSs are prevalent in older individuals and patients with cerebrovascular disease; as a result, most previous studies on EPVSs have been restricted to individuals with geriatric or cerebrovascular disease [18, 19]. Research on EPVSs in the neonatal period, particularly in brain injuries linked to PA, is in its infancy. Further studies are clearly warranted.

The primary objective of this study was to investigate whether EPVSs can serve as a novel imaging biomarker for the assessment of PABI severity and to explore correlations between the number of EPVs and clinical parameters. In addition, we aimed to explore the potential for EPVSs to predict later sleep outcomes in infants with PA. By evaluating correlations between EPVSs and clinical parameters, the severity of brain injury and sleep outcomes, this study will provide valuable insights and potential tools for the early diagnosis, prognosis, and intervention of neonatal brain injury.

## Methods

### Selection of participants

**Fig. 1:**
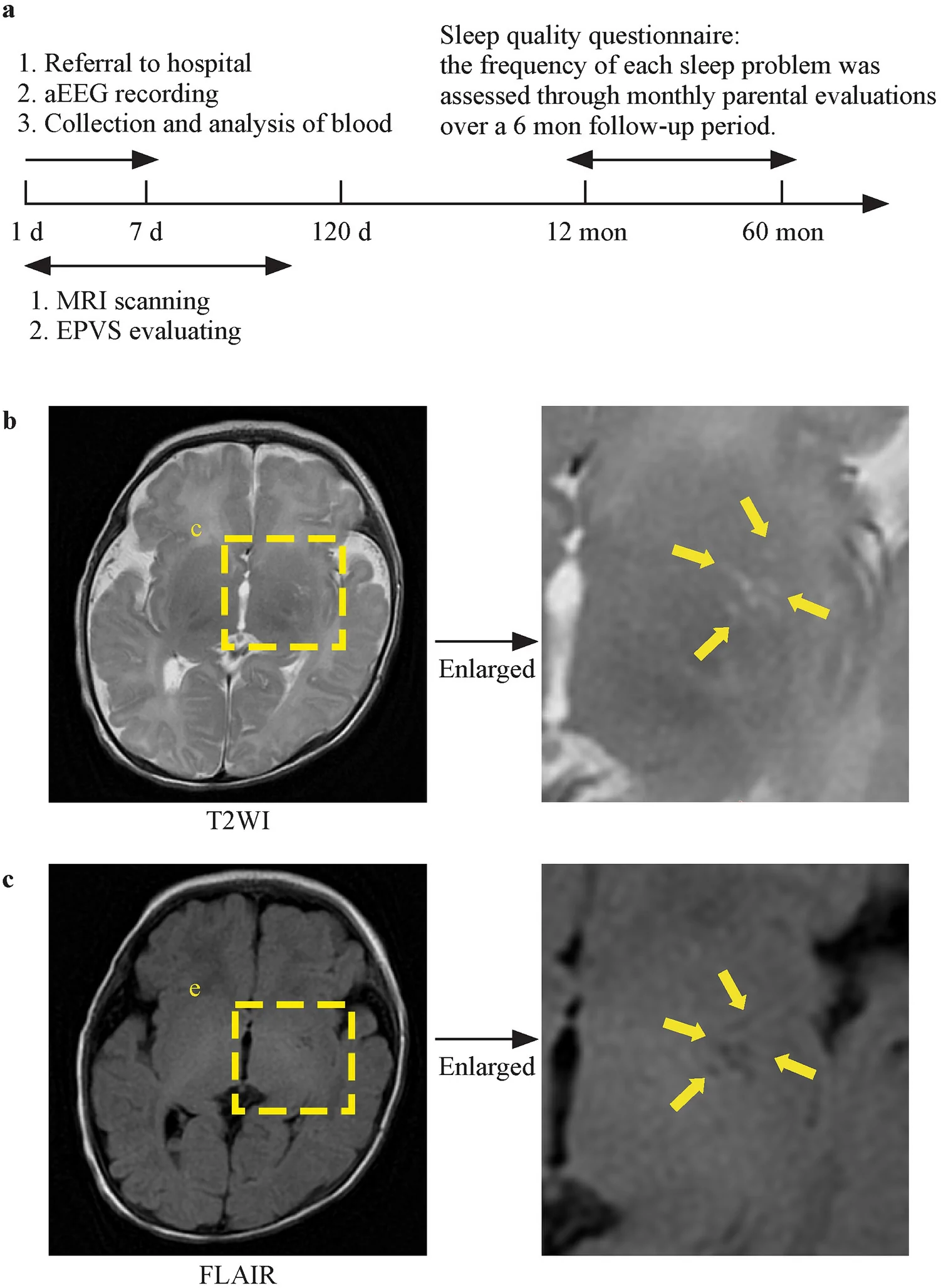
Patient evaluation workflow and representative MRI images. **a** Workflow for patient evaluation following hospital admission; **b** T2W images; **c** FLAIR images. *T2WI* T2-weighted imaging, *FLAIR* fluid attenuated inversion recovery, *MRI* magnetic resonance imaging, *aEEG* amplitude-integrated electroencephalography, *EPVS* enlarged perivascular spaces

We included newborns with PA admitted to the Department of Neonatology, Children’s Hospital of Soochow University, between January 2020 and September 2023. All the newborns were transferred to our hospital within one week of birth. Workflow for procedures performed after patient transfer can be found in Fig. 1a. Exclusion criteria were as follows: (1) a history of traumatic brain injury, intracranial infection, stroke, or other non-asphyxia-related intracranial diseases; (2) major congenital malformations (e.g., complex congenital heart disease, severe central nervous system structural abnormalities) or known genetic syndromes; (3) a maternal history of long-term substance abuse (e.g., alcohol, drugs), uncontrolled diabetes or thyroid disease or other underlying diseases; (4) poor-quality cranial magnetic resonance (MR) images rendering accurate EPVS assessment impossible; (5) missing clinical data.

Diagnosis of PA was followed the strict criteria for neonatal asphyxia. Disease severity was graded based on the Apgar score and the results of postnatal umbilical artery blood gas analysis [20]. Newborns were divided into groups with mild or severe asphyxia. Mild PA was defined on the basis of an Apgar score of 4–7 at one minute or an Apgar score of 4–7 at five minutes and/or an umbilical artery pH < 7.20. Severe asphyxia was defined on the basis of an Apgar score of 0–3 at one minute or an Apgar score of 0–5 at five minutes and/or an umbilical artery pH < 7.00. Follow-up sleep assessments were completed for 258 of the 319 enrolled newborns, including 182 in the mild and 76 in the severe PA group. Families of the remaining 61 newborns declined participation or were unreachable. The study protocol was approved by the Ethics Committee of Children’ s Hospital of Soochow University (approval No. 2017038). Written informed consent was obtained from all participants’ guardians.

### Magnetic resonance imaging acquisition and evaluation

All the subjects underwent magnetic resonance imaging (MRI) scanning using a GE Discovery MR750w 3.0 T scanner. All sequences had a slice thickness of 5 mm. T1-weighted imaging (T1WI) parameters were as follows: repetition time = 2500 ms, echo time = 31 ms, and field of view (FOV) = 512 × 512 mm^2^. T2-weighted imaging (T2WI) parameters were as follows: repetition time = 5100 ms, echo time = 103 ms, and FOV = 512 × 512 mm^2^. Fluid-attenuated inversion recovery (FLAIR) parameters were as follows: repetition time = 9800 ms, echo time = 92 ms, and FOV = 512 × 512 mm^2^. Diffusion-weighted imaging (DWI) parameters were as follows: repetition time = 4300 ms, echo time = 75 ms, FOV = 256 × 256 mm^2^, b-value = 750 s/mm^2^. Using these MR images, a simplified MRI score was given to each newborn [21, 22]. Severity was classified from grades 0–3 as follows: (1) grade 0, normal; (2) grade 1, signal abnormalities in one area (the watershed zone, basal ganglia/thalamus, posterior limb of internal capsule, brainstem); (3) grade 2, abnormalities involving two or three regions; (4) grade 3, extensive brain damage.

### Assessment of enlarged perivascular spaces number

Two experienced radiologists who were blinded to the clinical information assessed the number of EPVSs [23]. The PVS is filled with cerebrospinal fluid (CSF). The amount of CSF in the normal PVS is insufficient for detection; in cases with EPVSs, the CSF signal appears hyperintense on T2 images and hypointense on FLAIR images, linear when parallel to the vessel, and circular or elliptical when perpendicular (Fig. 1b–c). To quantify EPVSs in the basal ganglia, 4–6 axial sections through this region were reviewed from superior to inferior, and number of EPVSs in each section were counted and summed.

### Amplitude-integrated electroencephalography recording and evaluation

A pair of bilateral parietal bone electrodes was placed at P3/P4 for aEEG recording (Natus 580-NLICU1, USA). aEEG recordings were initiated after admission and on the same day as MRI. Background patterns, sleep-wake cycles (SWCs), and epileptic seizures were assessed. On the basis of these three factors [24, 25], aEEG was conventionally graded into three grades: (1) grade 0, normal: a continuous normal amplitude, an age-appropriate SWC, and no electrographic seizures; (2) grade 1, mildly abnormal: discontinuous activity, a mildly abnormal amplitude, an immature or delayed SWC, or normal amplitude with electrographic seizures; and (3) grade 2, severely abnormal: discontinuous activity; a severely abnormal amplitude; no SWC [including burst-suppression, flat tracing (electrical silence), persistent low voltage]; or a mildly abnormal amplitude with electrographic seizures.

### Patient demographic and laboratory test data

Basic demographic and clinical information, including gestational age, birth weight, and head circumference, was obtained from the electronic hospital information system of the Children’s Hospital of Soochow University. Perinatal data, including Apgar scores at 1, 5, and 10 minutes, were retrieved from delivery room records and neonatal resuscitation documentation. Venous blood samples were collected within 24 hours of admission and sent to the laboratory for routine blood tests, blood gas analysis, and coagulation tests.

### Sleep quality questionnaire

Based on the Brief Infant Sleep Questionnaire, the Paediatric Sleep Questionnaire and previous studies [23, 24, 26, 27], the following sleep-related problems were assessed using these questions: (1) a variable start-of-night sleep time; (2) a prolonged time to fall asleep (night-time sleep latency exceeding 20 min); (3) insufficient sleep (fewer than 11 h); (4) variability in daily sleep schedule (fluctuations in nap start time, duration, and night-time sleep length); (5) loud snoring; (6) sleep-related breathing problems (rapid breathing and intermittent apnoea during sleep); and (7) frequent night-time awakenings (more than twice per night). The frequency of each problem was assessed through monthly parental evaluations over a 6-month follow-up period. Each sleep-related problem was scored as follows: a score of 0 was assigned if the problem did not occur every month, a score of one was assigned if the problem occurred every month but no more than 3 times during the most frequent week of each month, and a score of two was assigned if the problem occurred every month and more than three times during any single week of any month. The total sleep score was calculated as the sum of all item scores.

### Statistical analysis

Data were analysed using GraphPad Prism (GraphPad Inc., San Diego, CA, USA, version 9) and R software (R Foundation, version 4.4.11). Normal distribution for all continuous variables was assessed using the Shapiro-Wilk test. All data are presented as the mean ± standard deviation (SD), with the median (25th percentile, 75th percentile) additionally reported for non-normally distributed variables. For comparisons between two groups, Student’s* t* test was used for normally distributed data, and the Mann-Whitney *U* test was used for non-normally distributed data. Comparisons of sleep scores among patients in different age groups was performed using the Kruskal-Wallis test. All correlation analyses were performed using Spearman’s rank correlation, with *r* and *P* values used to indicate correlation strength and significance, respectively. Multivariable linear regression models were constructed to analyse the independent correlations between the number of EPVSs and the severity of brain injury. Covariates were selected based on univariate association (*P* < 0.05) and biological plausibility. A *P* value < 0.05 indicates statistical significance.

## Results

### Baseline participant characteristics

A total of 319 neonates were included, with 229 in the mild PA group (102 males and 127 females) and 90 in the severe PA group (47 males and 43 females). Table 1 shows the detailed demographic and clinical characteristics of all the study participants. No significant differences were found in head circumference, heart rate, white blood cell count (WBC), red blood cell count (RBC), procalcitonin (PCT), arterial partial pressure of carbon dioxide (PaCO₂), or pH between the mild and severe PA groups. However, the interval from birth to MRI was significantly longer in severe PA group. Compared with the severe PA group, the mild PA group had significantly higher gestational age, birth weight, systolic and diastolic blood pressures, Apgar scores at 1, 5, and 10 minutes, platelet count, PaO₂, and base excess (BE). Conversely, lactate levels, C-reactive protein (CRP) levels, and number of EPVSs were significantly higher in the severe PA group.

**Table 1:** Baseline participant characteristics

Characteristics	Total (*n* = 319)	Mild PA group (*n* = 229)	Severe PA group (*n* = 90)	*P* value
Physiological				
Gestational age (d)	259.0 (230.0, 278.0)	263.0 (233.0, 280.0)	254.0 (218.0, 272.0)	0.0033
Interval from birth to MRI scanning (d)	11.0 (8.0, 23.0)	10.0 (8.0, 19.0)	14.0 (10.0, 29.0)	0.0008
Weight of birth (g)	2750.0 (1810.0, 3350.0)	2900.0 (1915.0, 3470.0)	2393.2 ± 883.8	0.0013
Head circumference (cm)	33.0 (30.0, 34.0)	33.0 (30.0, 34.5)	32.5 (29.0, 34.0)	0.0785
Heart rate (BPM)	138.0 (127.0, 145.0)	138.0 (128.0, 145.0)	135.0 (122.0, 145.0)	0.1618
Systolic pressure (mmHg)	64.0 (57.0, 72.0)	65.0 (58.0, 72.0)	61.2 ± 10.6	0.0018
Diastolic pressure (mmHg)	35.0 (30.0, 42.0)	37.4 ± 9.0	33.6 ± 9.0	0.0079
Apgar score				
1 min	6.0 (3.0, 7.0)	7.0 (6.0, 7.0)	2.0 (1.0, 3.0)	< 0.0001
5 min	8.0 (7.0, 9.0)	9.0 (8.0, 9.0)	6.0 (4.0, 8.0)	< 0.0001
10 min	10.0 (9.0, 10.0)	10.0 (9.0, 10.0)	8.0 (6.0, 10.0)	< 0.0001
Routine blood work				
WBC (10^9^/L)	15.5 (10.6, 20.5)	15.9 (10.6, 20.8)	14.2 (10.4, 19.9)	0.1935
RBC (10^12^/L)	4.6 (4.2, 5.0)	4.7 (4.2, 5.0)	4.5 ± 0.7	0.0767
PLT (10^9^/L)	238.0 (195.0, 278.0)	247.5 (207.5, 282.0)	220.3 ± 66.4	0.0007
CRP (mg/L)	0.5 (0.2, 0.5)	0.5 (0.2, 0.5)	0.5 (0.5, 0.5)	0.0117
PCT (ng/mL)	0.4 (0.2, 1.9)	0.4 (0.2, 1.6)	0.6 (0.2, 3.3)	0.1195
Blood gas data				
pH	7.4 (7.3, 7.5)	7.4 (7.3, 7.5)	7.4 (7.3,7.5)	0.0592
PaO_2_ (mmHg)	130.0 (92.0, 151.0)	136.0 (106.0, 154.0)	115.0 (79.0, 141.0)	< 0.0001
PaCO_2_ (mmHg)	27.9 (23.3, 35.2)	27.7 (23.9, 34.4)	28.6 (21.2, 36.5)	0.2706
BE (mmol/L)	−5.6 (−8.6,−4.5)	−5.5(−7.3,−3.9)	−6.3 (−9.3,−4.1)	0.0201
LAC (mmol/L)	4.5 (3.4, 6.9)	4.3 (2.9, 6.3)	5.2 (3.6, 10.8)	< 0.0001
EPVS				
EPVS number	4.0 (2.0, 8.0)	4.6 ± 4.2	7.0 (4.0, 11.0)	< 0.0001

Normal distribution was assessed using the Shapiro-Wilk test. Normally distributed data are presented as the mean ± standard deviation (SD), and non-normally distributed data are presented as the median (interquartile range, IQR). Group comparisons for non-normally distributed data were performed using the Mann-Whitney *U* test, whereas Student’s* t* test was used for normally distributed data. A *P* value < 0.05 indicates statistical significance

*PA* perinatal asphyxia, *MRI* magnetic resonance imaging, *BPM* beats per minute, *WBC* white blood cell count, *RBC* red blood cell count, *PLT* platelet count, *CRP* C-reactive protein, *PCT* procalcitonin, *PaO₂* arterial partial pressure of oxygen, *PaCO₂* arterial partial pressure of carbon dioxide, *BE* base excess, *LAC* lactate, *EPVS* enlarged perivascular spaces

### Correlation between enlarged perivascular spaces number and clinical measurements

**Fig. 2:**
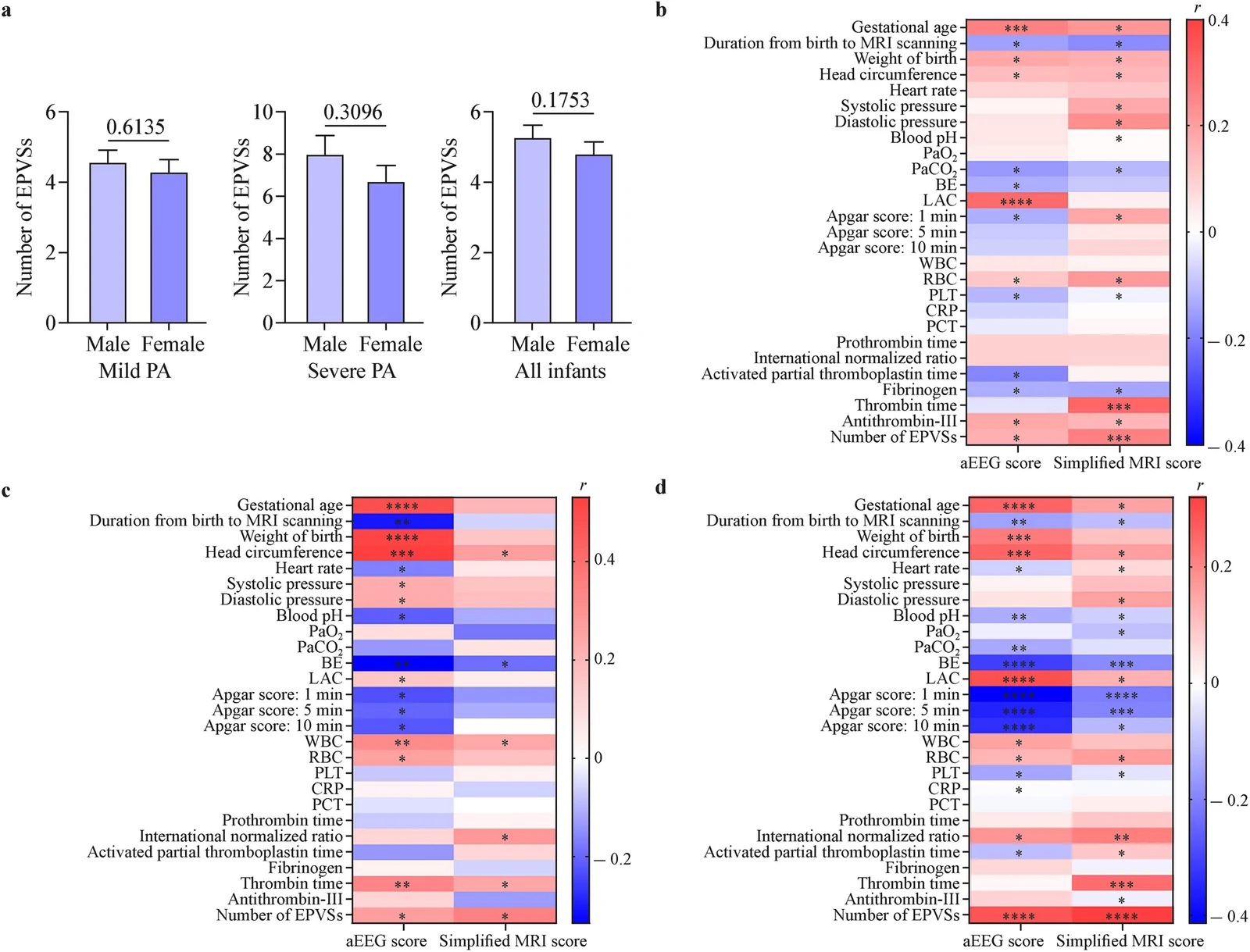
Comparison of EPVS number between sexes and correlations with clinical parameters and brain injury severity. **a** comparison of EPVSs number between sexes using Mann-Whitney *U* test;** b** correlation analysis between clinical indicators and aEEG and simplified MRI scores in the mild PA group; **c** correlation analysis between clinical indicators and aEEG and simplified MRI scores in the severe PA group; **d** correlation analysis between clinical indicators and aEEG and simplified MRI scores in all infants. In **b**–**d**, analyses were performed using Spearman’s rank correlation. Results were presented with *r* and *P* values. *P < 0.05, **P < 0.01, ***P < 0.001, ****P < 0.0001. *EPVS* enlarged perivascular spaces, *PA* perinatal asphyxia, *aEEG* amplitude-integrated electroencephalography, *WBC* white blood cell count, *RBC* red blood cell count, *PLT* platelet count, *CRP* C-reactive protein, *PCT* procalcitonin, *PaO₂* arterial partial pressure of oxygen, *PaCO₂* arterial partial pressure of carbon dioxide, *BE* base excess, *LAC* lactate

There were no significant sex-related differences in the number of EPVSs in the mild PA group, severe PA group, or all infants (Fig. 2a). Univariate analysis revealed that the number of EPVSs correlated positively with gestational age, birth weight, and head circumference in both the mild and severe PA groups; this was stronger in severe cases. In the mild PA group, the number of EPVSs also correlated with blood pressure and antithrombin-III levels and negatively correlated with postnatal age at MRI and 10-min Apgar score. In the severe PA group, the number of EPVSs correlated negatively with the 1-min Apgar score and positively with diastolic blood pressure. For all infants, the number of EPVSs correlated positively with diastolic blood pressure and lactate levels and negatively with Apgar score at all time points along with postnatal age at MRI. Inflammatory markers, coagulation parameters, and blood gas indices were not consistently associated with the number of EPVSs (Table 2).

**Table 2:** Correlations of numbers of EPVSs with clinical parameters

Characteristic	Mild PA group (*n* = 229)		Severe PA group (*n* = 90)		Total (*n* = 319)	
	*r*	*P* value	*r*	*P* value	*r*	*P* value
Physiological						
Gestational age (d)	0.244	0.0001	0.330	0.0008	0.218	< 0.0001
Interval from birth to MRI scanning (d)	− 0.138	0.0185	− 0.167	0.0589	− 0.114	0.0209
Weight of birth (g)	0.228	0.0003	0.328	0.0009	0.197	0.0002
Head circumference (cm)	0.202	0.0011	0.326	0.0009	0.214	< 0.0001
Heart rate (BPM)	− 0.005	0.4722	0.021	0.4228	− 0.020	0.3646
Systolic pressure (mmHg)	0.141	0.0168	0.126	0.1199	0.077	0.0860
Diastolic pressure (mmHg)	0.120	0.0356	0.265	0.0061	0.117	0.0188
Apgar score						
1 min	0.009	0.4473	− 0.219	0.0197	− 0.295	< 0.0001
5 min	− 0.024	0.3596	− 0.034	0.3749	− 0.205	0.0001
10 min	− 0.143	0.0154	0.065	0.2715	− 0.159	0.0022
Routine blood work						
WBC (10^9^/L)	0.092	0.0835	0.054	0.3077	0.071	0.1035
RBC (10^12^/L)	0.070	0.1467	0.168	0.0583	0.078	0.0840
PLT (10^9^/L)	0.055	0.2029	− 0.018	0.4333	− 0.017	0.3813
CRP (mg/L)	− 0.020	0.3682	− 0.073	0.2494	− 0.028	0.3115
PCT (ng/mL)	− 0.026	0.3457	0.053	0.3119	0.019	0.3671
Blood gas data						
pH	0.091	0.0849	− 0.043	0.3455	− 0.003	0.4784
PaO_2_ (mmHg)	0.047	0.2384	0.089	0.2030	0.004	0.4701
PaCO_2_ (mmHg)	− 0.135	0.0209	0.074	0.2441	− 0.069	0.1108
BE (mmol/L)	− 0.026	0.3503	− 0.012	0.4556	− 0.079	0.0802
LAC (mmol/L)	0.071	0.1435	0.008	0.4709	0.127	0.0120
Blood coagulation						
Prothrombin time (s)	− 0.020	0.3823	− 0.077	0.2372	0.0004	0.4972
INR	− 0.011	0.4310	0.0488	0.3246	0.070	0.1072
APTT (s)	− 0.094	0.0827	− 0.147	0.0852	− 0.078	0.0827
Fibrinogen (g/L)	− 0.082	0.1110	− 0.027	0.4018	0.006	0.4560
TT (s)	0.142	0.0164	0.010	0.4630	0.102	0.0347
AT3 (%)	0.191	0.0019	0.013	0.4508	0.072	0.1001

All correlation analyses were performed using Spearman's rank correlation. Correlations are presented with* r* and *P* values. A *P* value < 0.05 indicates statistical significance

*EPVS* enlarged perivascular spaces, *PA* perinatal asphyxia, *MRI* magnetic resonance imaging, *WBC* white blood cell count, *RBC* red blood cell count, *PLT* platelet count, *CRP* C-reactive protein, *PCT* procalcitonin, *PaO₂* arterial partial pressure of oxygen, *PaCO₂* arterial partial pressure of carbon dioxide, *BE* base excess, *LAC* lactate, *INR* international normalized ratio, *APTT* activated partial thromboplastin time, *TT* thrombin time, *AT3* antithrombin-III

### Correlation between enlarged perivascular spaces number and the severity of perinatal asphyxia-induced brain injury

The aEEG and simplified MRI scores described above were used as objective measures of the severity of PABI. There was a significant positive correlation between the number of EPVSs and both the aEEG and simplified MRI scores (Fig. 2d). Subgroup analysis of newborns with mild and severe PA also confirmed this significant positive correlation (Fig. 2b–c). In addition to the numbers of EPVSs, multiple clinical measurements were significantly correlated with the severity of PABI. In the full cohort, gestational age, thrombin time, and international normalized ratio (INR) were positively correlated with brain injury score, whereas base excess, Apgar scores, and platelet count were negatively correlated. Subgroup analyses revealed that in the mild PA group, thrombin time was positively correlated with the simplified MRI score; and APTT was negatively correlated with the aEEG score. In the severe PA group, gestational age, birth weight, head circumference, and blood pressure were positively correlated with aEEG score, while BE was negatively correlated with both the aEEG and simplified MRI scores.

Multivariate linear regression analysis was conducted to identify independent correlates of the aEEG score in all participants. Variables showing a significant univariate correlation were included as independent variables, while birth weight and head circumference were excluded because of their high collinearity with gestational age. Gestational age, BE, and the 1-min Apgar score were independently associated with the aEEG score. A multivariate linear regression model was also established with the simplified MRI score as the dependent variable. Variables that correlated with the simplified MRI score were included; head circumference was excluded because of collinearity with gestational age. Diastolic blood pressure, base excess, 5-min and 10-min Apgar scores, INR, thrombin time, and number of EPVSs were independently associated with the simplified MRI score (Table 3).

**Table 3:** Multivariate linear regression analysis of factors associated with the severity of brain injury in all infants

Variable	β	SE	95% CI	*P* value
All infants (aEEG)				
Gestational age (d)	0.0063	0.0021	0.0021 to 0.0105	0.0030
Interval from birth to MRI scanning (d)	0.0021	0.0026	− 0.0030 to 0.0072	0.4242
pH	0.0519	0.4508	− 0.8353 to 0.9390	0.9085
PaCO_2_ (mmHg)	− 0.0020	0.0044	− 0.0105 to 0.0066	0.6542
BE (mmol/L)	− 0.0294	0.0121	− 0.0531 to − 0.0056	0.0157
LAC (mmol/L)	− 0.0156	0.0116	− 0.0384 to 0.0073	0.1806
Apgar score (1 min)	− 0.0761	0.0182	− 0.1119 to − 0.0402	< 0.0001
Apgar score (5 min)	− 0.0225	0.0276	− 0.0768 to 0.0318	0.4147
Apgar score (10 min)	− 0.0348	0.0294	− 0.0926 to 0.0231	0.2383
WBC (10^9^/L)	−0.0018	0.0042	− 0.0101 to 0.0066	0.6786
RBC (10^12^/L)	0.0411	0.0469	− 0.0513 to 0.1334	0.3821
PLT (10^9^/L)	− 0.0007	0.0004	− 0.0016 to 0.0001	0.0869
INR	0.0826	0.0987	− 0.1117 to 0.2769	0.4034
APTT (s)	− 0.0014	0.0019	− 0.0052 to 0.0025	0.4887
EPVS number	0.0109	0.0067	− 0.0023 to 0.0240	0.1057
All infants (MRI)				
Gestational age (d)	0.0004	0.0018	− 0.0032 to 0.0040	0.8184
Interval from birth to MRI scanning (d)	− 0.0021	0.0024	− 0.0069 to 0.0028	0.3989
PaO_2_ (mmHg)	− 0.0009	0.0006	− 0.0021 to 0.0003	0.1554
Diastolic pressure (mmHg)	0.0075	0.0036	0.0004 to 0.0146	0.0394
BE (mmol/L)	− 0.0184	0.0078	− 0.0339 to − 0.0030	0.0192
LAC (mmol/L)	− 0.0180	0.0108	− 0.0393 to 0.0033	0.0979
Apgar score (1 min)	− 0.0246	0.0174	− 0.0589 to 0.0096	0.1583
Apgar score (5 min)	− 0.0639	0.0258	− 0.1146 to − 0.0133	0.0136
Apgar score (10 min)	0.0537	0.0272	0.0001 to 0.1073	0.0495
RBC (10^12^/L)	0.0354	0.0428	− 0.0489 to 0.1197	0.4090
PLT (10^9^/L)	0.0001	0.0004	− 0.0007 to 0.0009	0.7585
INR	0.1752	0.0845	0.0091 to 0.3414	0.0388
TT (s)	0.0061	0.0018	0.0026 to 0.0096	0.0007
EPVS number	0.0247	0.0064	0.0122 to 0.0372	0.0001

A *P* value < 0.05 indicates statistical significance

*β* regression coefficient, *95%CI* 95% confidence interval, *aEEG* amplitude-integrated electroencephalography, *MRI* magnetic resonance imaging, *BE* base excess, *LAC* lactate, *WBC* white blood cell count, *RBC* red blood cell count, *PLT* platelet count, *INR* international normalized ratio, *APTT* activated partial thromboplastin time, *PaO₂* arterial partial pressure of oxygen, *TT* thrombin time, *EPVS* enlarged perivascular spaces

Subgroup analyses were subsequently performed. In the mild PA group, weight after birth and head circumference were excluded to avoid multicollinearity; lactate was identified as the sole independent predictor of the aEEG score. Number of EPVSs were not independently correlated. In addition, in this group, thrombin time and number of EPVSs were independently associated with the simplified MRI score. In the severe PA group, gestational age was the only independent correlate of the aEEG score, with number of EPVSs showing no significant association. Thrombin time and number of EPVSs remained independently associated with the simplified MRI score (Tables 4 and 5).

**Table 4:** Multivariate linear regression analysis of factors associated with the severity of brain injury in the mild PA group

Variable	Estimate	Standard error	95% CI	*P* value
Mild PA group (aEEG)				
Gestational age (d)	0.0004	0.0021	− 0.0037 to 0.0045	0.8515
Interval from birth to MRI scanning (d)	0.0005	0.0024	− 0.0043 to 0.0053	0.8352
RBC (10^12^/L)	0.0519	0.0412	− 0.0293 to 0.1330	0.2093
PLT (10^9^/L)	− 0.0005	0.0004	− 0.0012 to 0.0003	0.2089
APTT (s)	− 0.0023	0.0019	− 0.0060 to 0.0014	0.2178
FT (g/L)	− 0.0756	0.0410	− 0.1563 to 0.0052	0.0664
AT3 (%)	0.0031	0.0032	− 0.0031 to 0.0094	0.3222
Apgar score (1 min)	− 0.0344	0.0246	− 0.0829 to 0.0140	0.1627
PaCO_2_ (mmHg)	− 0.0002	0.0028	− 0.0057 to 0.0053	0.9353
BE (mmol/L)	0.0020	0.0105	− 0.0187 to 0.0227	0.8475
LAC (mmol/L)	0.0336	0.0135	0.0070 to 0.0603	0.0135
EPVS number	0.0077	0.0066	− 0.0054 to 0.0207	0.2465
Mild PA group (MRI)				
Gestational age (d)	− 0.0010	0.0020	− 0.0050 to 0.0030	0.6118
Interval from birth to MRI scanning (d)	− 0.0025	0.0024	− 0.0073 to 0.0024	0.3156
Systolic pressure (mmHg)	− 0.0002	0.0043	− 0.0087 to 0.0083	0.9605
Diastolic pressure (mmHg)	0.0057	0.0048	− 0.0037 to 0.0151	0.2336
PaCO_2_ (mmHg)	− 0.0033	0.0027	− 0.0086 to 0.0020	0.2247
Apgar score (1 min)	0.0476	0.0251	− 0.0019 to 0.0970	0.0594
RBC (10^12^/L)	0.0452	0.0414	− 0.0363 to 0.1268	0.2755
FT (g/L)	− 0.0466	0.0405	− 0.1265 to 0.0332	0.2511
TT (s)	0.0062	0.0015	0.0033 to 0.0092	< 0.0001
AT3 (%)	0.0034	0.0032	− 0.0029 to 0.0096	0.2937
EPVS number	0.0156	0.0069	0.0020 to 0.0292	0.0248

A* P* value < 0.05 indicates statistical significance

*PA* perinatal asphyxia, *aEEG* amplitude-integrated electroencephalography, *MRI* magnetic resonance imaging, *RBC* red blood cell count, *PLT* platelet count, *APTT* activated partial thromboplastin time, *FT* fibrinogen, *AT3* antithrombin-III, *PaCO₂* arterial partial pressure of carbon dioxide, *BE* base excess, *LAC* lactate, *TT* thrombin time, *EPVS* enlarged perivascular spaces

**Table 5:** Multivariate linear regression analysis of factors associated with the severity of brain injury in the severe PA group

Variable	Estimate	Standard error	95% CI	*P* value
Severe PA group (aEEG)				
Gestational age (d)	0.0055	0.0019	0.0017 to 0.0092	0.0042
Interval from birth to MRI scanning (d)	0.0024	0.0024	− 0.0024 to 0.0071	0.3305
Systolic pressure (mmHg)	− 0.0066	0.0042	− 0.0149 to 0.0018	0.1234
Diastolic pressure (mmHg)	0.0014	0.0047	− 0.0078 to 0.0107	0.7590
pH	0.3236	0.2890	− 0.2461 to 0.8933	0.2641
BE (mmol/L)	− 0.0177	0.0096	− 0.0366 to 0.0011	0.0653
Apgar score (1 min)	− 0.0415	0.0246	− 0.0900 to 0.0071	0.0938
Apgar score (5 min)	− 0.0099	0.0308	− 0.0706 to 0.0508	0.7483
Apgar score (10 min)	− 0.0135	0.0395	− 0.0914 to 0.0645	0.7337
WBC (10^9^/L)	− 0.0060	0.0040	− 0.0139 to 0.0019	0.1360
RBC (10^12^/L)	0.0303	0.0408	− 0.0501 to 0.1107	0.4582
TT (s)	− 0.0012	0.0015	− 0.0041 to 0.0017	0.4140
EPVS number	0.0075	0.0069	− 0.0060 to 0.0210	0.2730
Severe PA group (MRI)				
Head circumference (cm)	0.0169	0.0090	− 0.0007 to 0.0345	0.0605
BE (mmol/L)	− 0.0096	0.0090	− 0.0273 to 0.0081	0.2853
WBC (10^9^/L)	− 0.0003	0.0039	− 0.0080 to 0.0074	0.9372
INR	0.0408	0.0851	− 0.1271 to 0.2086	0.6326
TT (s)	0.0065	0.0015	0.0035 to 0.0095	< 0.0001
EPVS number	0.0176	0.0069	0.0040 to 0.0312	0.0114

A *P* value < 0.05 indicates statistical significance

*PA* perinatal asphyxia, *aEEG* amplitude-integrated electroencephalography, *MRI* magnetic resonance imaging, *BE* base excess, *WBC* white blood cell count, *RBC* red blood cell count, *INR* international normalized ratio, *TT* thrombin time, *EPVS* enlarged perivascular spaces

### Correlation between enlarged perivascular spaces number and future sleep quality

**Fig. 3:**
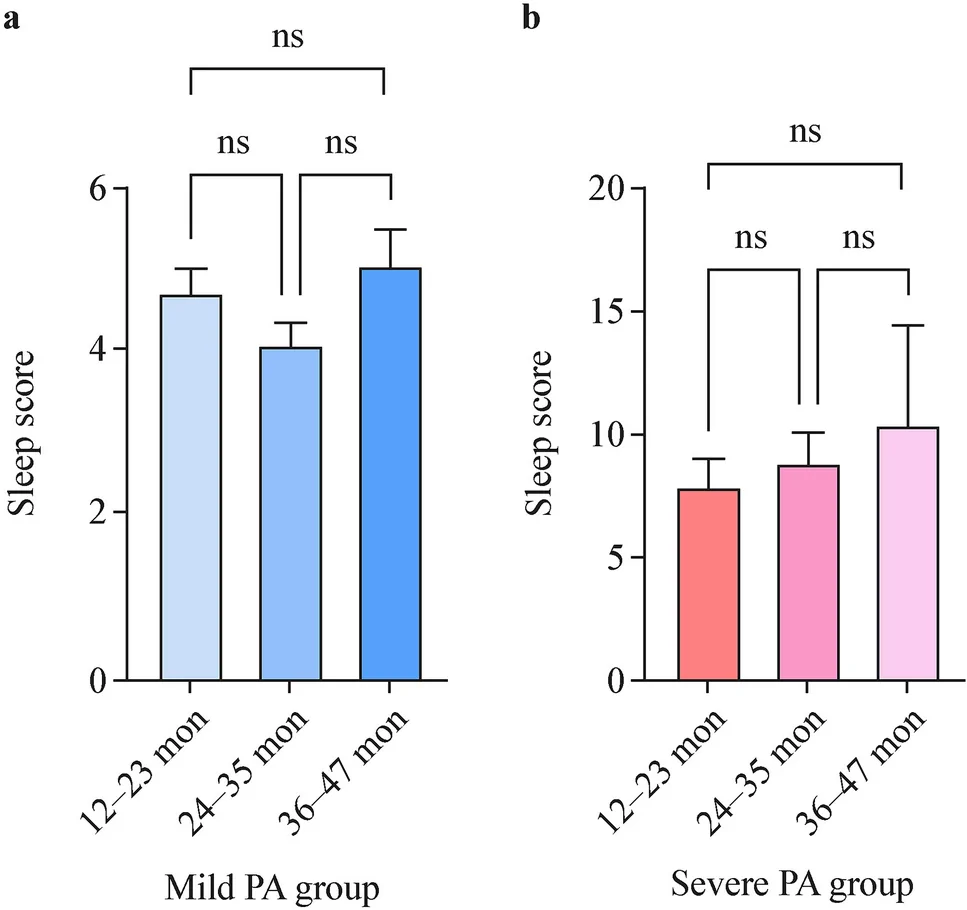
Comparison of sleep scores during follow-up among different age groups using Kruskal-Wallis test. **a** Mild PA group; **b** Severe PA group. *PA* perinatal asphyxia, *ns* no significant

**Fig. 4:**
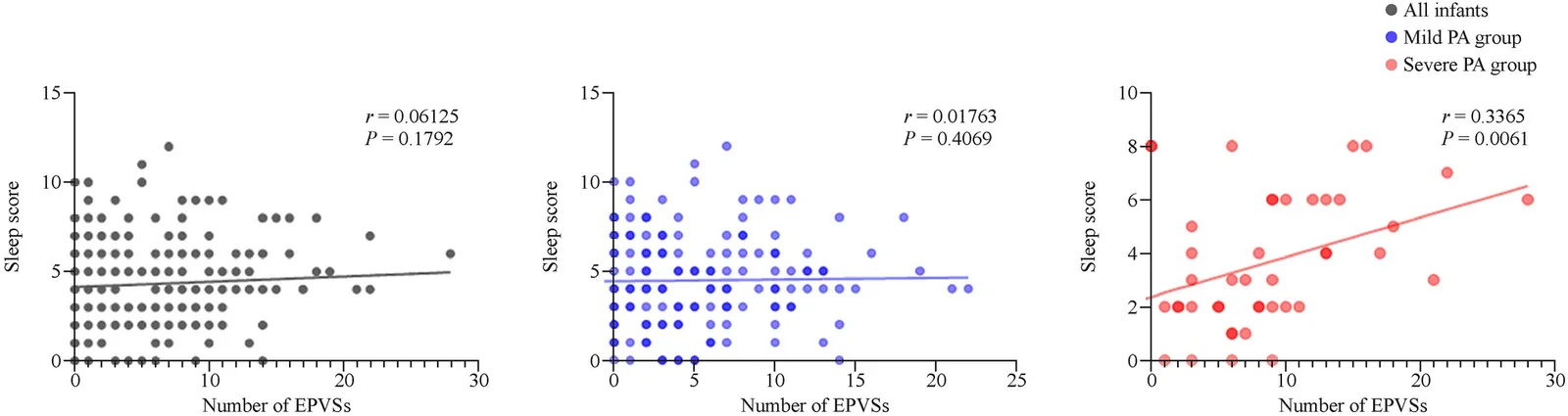
Correlation between sleep scores and number of EPVSs. Analyses were performed using Spearman’s rank correlation. *P* value < 0.05 indicates statistical significance. *PA* perinatal asphyxia, *EPVS* enlarged perivascular spaces

Follow-up sleep quality assessments were completed for 258 of the 319 enrolled infants (81.1%), including 182 in the mild PA group and 76 in the severe PA group. Families of the remaining 61 patients either declined participation or were unreachable. Amongst the 258 infants who completed follow-up, 227 were aged ≥ 12 months and were included in the sleep quality analysis (181 in the mild PA group and 46 in the severe PA group). To exclude age as a confounding factor, infants were stratified into three age groups (12–23 months, 24–35 months, and 36–47 months). No significant differences in sleep score were observed among these age subgroups within either the mild or severe PA group (Fig. 3a and b), supporting the pooling of all ages for subsequent correlation analyses. There was no significant correlation between sleep score and number of EPVSs in all newborns with PA or in the mild PA group. However, there was a significant positive correlation in the severe PA group (Fig. 4), indicating that a higher number of EPVSs was associated with more severe sleep disturbance.

## Discussion

In this study, we demonstrated that the number of EPVSs was independently associated with MRI-based severity of PABI and, in the severe PA subgroup, significantly correlated with later sleep disturbances. These findings position EPVS count as a candidate structural imaging biomarker that captures perivascular and glymphatic damage, a dimension not reflected by routine clinical or electrophysiological assessments. Given the insidious and often progressive nature of neonatal PA-induced brain injury [25], an accessible MRI marker directly linked to glymphatic dysfunction could facilitate earlier risk stratification. Mechanistically, asphyxia-triggered oxidative stress and inflammation disrupt the polarized expression of aquaporin 4 (AQP4) on astrocytic endfeet, leading to PVS enlargement and impaired glymphatic clearance [28, 29]. This glymphatic failure is not merely a consequence of injury; accumulating neurotoxic metabolites can further amplify inflammation and astrocytic injury, thereby perpetuating a vicious cycle of progressive brain damage. However, because the current study is observational, we cannot infer causality; EPVS may directly contribute to injury or represent an epiphenomenon of underlying pathology. Furthermore, the specificity of EPVS for PABI remains to be established against other neonatal encephalopathies, as perinatal stress and developmental immaturity could independently influence PVS visibility. Prospective cohort studies and experimental models are therefore needed to validate EPVS as a mechanistic biomarker and to define its clinical utility.

Of note, in multivariable models, EPVS number was independently associated with the simplified MRI score but not with the aEEG score. This dissociation reinforces the notion that EPVS primarily reflects structural rather than functional brain pathology. aEEG captures acute cortical electrical activity, which may be transiently suppressed by metabolic or ischemic insults; MRI, by contrast, visualizes established parenchymal injury. EPVS, as a marker of glymphatic structural integrity, aligns more closely with the latter. However, this selective association does not necessarily imply that EPVS is unrelated to functional outcomes, the relationship may be indirect or delayed, emerging only after structural damage reaches a certain threshold. Moreover, the cross-sectional design precludes conclusions about whether EPVS precedes, coincides with, or results from MRI-visible injury. Although the mechanisms of PABI (hypoxia, excitotoxicity, neuroinflammation, and oxidative stress) substantially overlap with those driving EPVS formation, both converging on astrocytic stress and loss of aquaporin-4 polarity [28, 29], this mechanistic overlap also introduces ambiguity: EPVS could be a parallel consequence of shared upstream pathways rather than an independent contributor to injury severity. Impaired cerebrovascular autoregulation and endothelial dysfunction may further confound this relationship by simultaneously promoting both brain injury and perivascular space enlargement [17, 30]. Therefore, while the independent association with MRI score supports the integration of EPVS count into multimodal assessment, the precise role of EPVS in disease progression remains to be defined. Longitudinal studies with serial imaging are required to determine whether EPVS enlargement predates overt parenchymal damage, and experimental models are indispensable to test whether interventions that preserve glymphatic function can reduce EPVS numbers and improve neurological outcomes.

Prior studies have attributed PA-related sleep problems primarily to pineal gland damage [31, 32]. Our data show that the number of EPVSs, particularly in severe PA, correlates with sleep score. This suggests that an additional mechanism beyond direct pineal injury is involved. Glymphatic damage leads to the accumulation of toxic proteins, creating a vicious cycle that exacerbates ongoing brain injury and indirectly impairs subsequent sleep quality [33]. Crucially, the predictive value of the number of EPVSs for sleep quality was significant only in the severe PA group, underscoring a severity-specific nature of this correlation. Although moderate, this correlation is biologically meaningful in the context of severe injury, in which glymphatic recovery is impeded. The number of EPVSs decreased in mild PA cases over time, reflecting a capacity to repair and explaining the lack of a correlation between the number of EPVSs and sleep quality. Infant sleep is multifactorial and cannot be explained by a single biomarker [34–36]. This study confirmed the absence of infant age or gestational age confounding and supports the specificity of this association. Future studies involving larger cohorts and stratified analyses are warranted.

Limitations of the study include the ethically driven absence of healthy controls. However, published data confirm the rarity of EPVSs in healthy neonates [37], supporting the pathological significance of EPVS observed in our asphyxiated cohort. Nevertheless, without a directly comparable control group, we cannot establish the precise threshold distinguishing physiological from pathological EPVS in neonates; our findings therefore require cautious interpretation. Other limitations include moderate sample size, single-centre design, and parent-reported sleep questionnaires. Future studies should incorporate healthy neonate controls where ethically feasible, employ objective sleep monitoring, and utilize multicentre cohorts with DTI-ALPS (diffusion tensor imaging along the perivascular space) imaging and longitudinal follow-up to validate EPVS as a prognostic biomarker.

In conclusion, EPVSs, as a non-invasive imaging marker, has unique value in evaluating PABI severity and predicting long-term sleep disturbances. Integration of the numbers of EPVSs into multimodal neonatal assessments may improve risk stratification and individualized intervention.

## Data Availability

The original data can be obtained from Xin Ding upon reasonable request.

## Declarations

### Declaration of AI Use

A large language model (Kimi, version 2.5) was employed for language improvement. During the preparation of this manuscript, the authors used generative artificial intelligence tools solely for language polishing and stylistic refinement. All academic ideas, research design, data analysis, and conclusions were independently completed by the authors and have been thoroughly reviewed. The authors take full responsibility for the accuracy, integrity, and scientific validity of all content in this paper.
